# *CFTR* Variant Frequencies and Newborn Screening Panel Performance in the Diverse CF Population Receiving Care in the State of Georgia

**DOI:** 10.3390/ijns11040085

**Published:** 2025-09-26

**Authors:** Eileen Barr, Brittany Truitt, Andrew Jergel, Shasha Bai, Kathleen McKie, Rossana Sanchez Russo, Kathryn E. Oliver, Rachel W. Linnemann

**Affiliations:** 1 Department of Human Genetics, Emory University, Atlanta, GA 30322, USArossana.sanchez@emory.edu (R.S.R.); 2 Children’s Healthcare of Atlanta, Atlanta, GA 30329, USA; brittany.ashton.truitt@emory.edu; 3 Department of Pediatrics, Emory University, Atlanta, GA 30322, USA; andrew.jergel@emory.edu (A.J.); shasha.bai@emory.edu (S.B.); kolive3@emory.edu (K.E.O.); 4 Department of Pediatrics, Augusta University, Augusta, GA 30912, USA; ktmckie@augusta.edu

**Keywords:** cystic fibrosis, newborn screening, cystic fibrosis transmembrane conductance regulator, case detection, DNA panel, health equity

## Abstract

Cystic fibrosis (CF) newborn screening (NBS) aims to improve outcomes through early diagnosis, yet disparities in time to diagnosis remain. This study examines *CFTR* allele frequencies and variant panel performance among a diverse CF population in Georgia to inform recommendations for updating the NBS algorithm and improving equity. This cross-sectional study includes 969 people with CF (PwCF) from Georgia’s accredited CF centers. *CFTR* variant frequencies were calculated according to race and ethnicity. Panel performance was evaluated for Georgia’s current Luminex-39 variant test and three expanded panels. Statistical analyses compared detection rates across panels and demographic groups. Georgia’s diverse CF population demonstrates a unique *CFTR* allelic variability compared to national data. Increasing panel size enhances case identification. A panel including 719 CF-causing variants from the CFTR2 database significantly improves case detection from 93% to 97% (*p* = 0.002), as well as two-variant detection from 69% to 86% (*p* < 0.001). Detection of minoritized PwCF also improves with increasing panel size. However, even using the 719-variant panel, detection of non-Hispanic Black PwCF remains significantly lower compared to non-Hispanic White PwCF (case detection: *p* = 0.003; two-variant detection: *p* < 0.001). In conclusion, the use of expanded *CFTR* panels for NBS in Georgia would enhance timely diagnosis and improve equity.

## 1. Introduction

Cystic fibrosis (CF) is a chronic, multi-system, genetic disorder that results in progressive lung disease and early mortality [1]. CF is caused by pathogenic variants in the CF transmembrane conductance regulator gene (*CFTR*), resulting in protein dysfunction [2]. Implementation of CF newborn screening (NBS) in the United States (U.S.) has led to diagnosis of most infants by 60 days of age [3,4]. Due to timely interventions, NBS results in superior outcomes such as enhanced nutrition, more rapid increase in lung function, and increased survival [4,5,6].

CF NBS programs strive to achieve high sensitivity by optimizing identification of true positive cases and minimizing false negative cases. CF NBS algorithms commence with quantification of immunoreactive trypsinogen (IRT) concentrations in dried blood spots from newborns, with an elevated IRT reflexing to either a repeat IRT measurement or *CFTR* variant screening, depending on the state [7]. The state of Georgia began CF NBS in 2007. The current algorithm employs an IRT value greater than or equal to a fixed cut-off of 55 ng/mL to trigger additional testing. Georgia’s second-tier test is a 39-variant *CFTR* variant panel (Luminex xTAG^®^ CF39v2), which is known to exhibit lower detection rates of pathogenic variants among minoritized individuals [8]. If the Luminex-39 test identifies at least one *CFTR* variant, the screen is considered positive. Infants with one variant detected are referred for sweat testing, whereas infants with two identified variants are referred directly to a CF Foundation-accredited care center. Infants who have positive or intermediate sweat test results are referred to a CF center for expanded genetic testing. All infants diagnosed with CF or CFTR-related metabolic syndrome are followed longitudinally at an accredited CF center.

Universal CF NBS has been performed by all 50 states since 2010, though genetic testing was not adopted by all states until 2020 [9,10,11]. Types of genetic tests vary by state—e.g., single variant screens, small panels of common variants, and next generation sequencing (NGS) of targeted *CFTR* variants [7]. A limited number of states have incorporated full *CFTR* sequence analysis using either traditional Sanger sequencing or the more comprehensive, higher-throughput NGS [9,12,13]. This strategy has the potential to expedite diagnosis for infants with rare variants and reduce the risk of missing CF infants with two uncommon variants [9,14,15,16,17,18]. Detection of heterozygotes and variants of varying clinical consequences must also be considered when contemplating larger variant panels and NGS. Higher heterozygote detection rates and uncertain clinical diagnoses can lead to increased burden on NBS follow-up programs and providers, as well as stress on patient families [19,20,21].

CF occurs in people of all racial and ethnic groups, yet individuals from minoritized groups face disparities in CF health outcomes [22,23]. These inequities begin early in life, with diagnostic delays occurring more often among minoritized infants and negatively impacting short- and long-term nutritional outcomes [24,25]. These delays may occur in part due to inequitable *CFTR* variant identification in state NBS algorithms that use common variant panels [9,11,15,17,26,27]. The state of Georgia has a higher Black/African American population than the national average, together with a sizeable Hispanic/Latino population [28]. The growing percentage of Black, Hispanic, and other non-White people with CF (PwCF) over the last 15 years [3], together with the increasing diversity of Georgia’s overall population, support the importance of investigating CF NBS approaches to more equitably provide benefits of early diagnosis to all infants. Therefore, this study aimed to examine *CFTR* variant frequencies and panel performance among a diverse CF population in Georgia to inform recommendations for updates to the state’s CF NBS algorithm to improve equity.

## 2. Materials and Methods

### 2.1. Study Design

This cross-sectional study included all individuals with a diagnosis of CF who were actively receiving care in 2022 at one of the two CF Foundation (CFF)-accredited care centers in Georgia: the Children’s Healthcare of Atlanta and Emory University Center or the Augusta University Center. Pediatric and adult PwCF were included. At each site, *CFTR* variants, race, and ethnicity were collected from our centers’ CFF Patient Registries (CFFPR). Enrollment and data collection for CFFPR has been described previously [29]. Missing data and imprecise variant annotation were reconciled through interrogation of electronic medical records and review of original genetic reports at each site, when available. Every individual in the CF cohort had undergone genetic testing. Race and ethnicity data were self-reported and entered in the medical record when a PwCF established care at the clinic. Those data were then submitted to the CFFPR by trained research coordinators. Race categories within CFFPR are American Indian or Alaskan Native, Asian, Black or African American, Native Hawaiian or Other Pacific Islander, Other/Unknown, Two or More Races, and White. Ethnicity is categorized as Hispanic/Latino, non-Hispanic/Latino, and Unknown. All study procedures were approved by the Emory University and Augusta University Institutional Review Boards (Emory IRB #00005155, Augusta IRB #1980623-4).

### 2.2. Race, Ethnicity, and CFTR Genotype Analyses

We compared the CF population in Georgia with the national U.S. CF population according to race and ethnicity. Data on the national CF population was obtained from the 2021 U.S. CF Foundation Patient Registry Annual Data Report [30]. In subsequent analyses, race and ethnicity variables were integrated such that individuals were not duplicated (White, non-Hispanic; Black or African American, non-Hispanic; Asian, non-Hispanic; Two or More races, non-Hispanic; Other, non-Hispanic; and Hispanic/Latino). Asian, Other, Two or More Races, American Indian or Alaskan Native, and Native Hawaiian or Other Pacific Islander were combined in some analyses due to insufficient sample size for comparisons. We then described PwCF in Georgia by genotype group (F508del homozygotes, F508del heterozygotes, no F508del variants) and by completeness of genotyping (Two Or More known variants, only one known variant, unknown variants) according to race and ethnicity. Variants classified as benign polymorphisms (7T, 9T, T854T, M470V, V470M) were categorized as unknown. Prevalence of the most common *CFTR* variants at the individual level was compared between PwCF in Georgia versus the national registry [30].

Variant frequency in Georgia (allele level) was established by counting the number of times a variant appeared in our dataset (some individuals had three variants). *CFTR* variant legacy names were used to align with nomenclature commonly seen on genetic testing panels and in the literature (Table S1). To include all variants that occurred more than five times, the 29 most common variants were listed. *CFTR* variants presently classified as benign (7T, 9T, T854T, M470V, V470M) were excluded from this analysis [31,32]. We then examined the frequency of the 29 most common variants in each racial/ethnic group. We also analyzed the five most prevalent *CFTR* alleles other than F508del that occurred within each racial/ethnic group, highlighting those not among the most common variants in Georgia and/or not currently included in the Luminex-39 panel.

### 2.3. Panel Comparison Analyses

*CFTR* panels commonly employed across various CF NBS laboratories were selected for variant detection analysis: Luminex-39, the current Georgia NBS panel (39 variants); Luminex-71 (71 variants); Illumina-139 NGS (139 variants); and a hypothetical Clinical and Functional Translation of CFTR (CFTR2) NGS panel (719 variants) that included all variants in the CFTR2 database classified as CF-causing (as of the April 2023 update) [8,32,33]. The CFTR2 database provides interpretations and genotype/phenotype data for *CFTR* variants reported in the CF population. Evaluation of variants aligns with standards of ACMG variant classifications [32,34]. Instruments required to analyze samples prepared for Luminex or Illumina platforms were already available at the Georgia Department of Public Health (DPH) laboratory. The CFTR2 panel was generated to evaluate performance of a test that incorporated the most comprehensive and up-to-date CF-causing variant list, similar to an approach taken for NBS in Wisconsin [35]. In late 2024, CFTR2 expanded the number of *CFTR* variants classified as disease-causing to 1085, and we replicated certain analyses with a hypothetical 1085-variant panel [32].

All people diagnosed with CF were included in the panel analyses, including those with one or two unknown (unidentified) variants. Each *CFTR* variant from an individual’s genotype was matched to the variant list from each panel. For detection rates of each panel, individuals were classified as having identification of one CF-causing variant, two variants (Two or More), or no variants. A variable for positive genetic screening rate (PGSR) was established by combining individuals with one detected variant that would trigger subsequent confirmatory sweat testing, together with those having two detected variants, to signify case identification. We then compared how detection rates for each panel differed between tests and by racial/ethnic group.

### 2.4. Statistical Methods

All variables were categorical and described with counts and percentages out of the total individuals or total alleles. Primary comparisons of interests were the overall and pairwise comparisons of PGSR by screening panels, as well as PGSR by race/ethnicity within each screening panel. All comparisons incorporated appropriate cell counts (≥5). Therefore, Pearson’s Chi-squared test and pairwise proportion test were used. For all pairwise comparisons of PGSR, non-Hispanic White was used as the reference for race/ethnicity to examine disparities, and Georgia’s current Luminex-39 variant panel was used as the panel reference. All pairwise comparisons were adjusted using Bonferroni correction to control for the overall type I error rates. For all comparisons, a *p*-value less than 0.05 was considered statistically significant. All data cleaning, graphics, and analyses were performed in R Statistical Software (v4.2.1; R Core Team 2022).

## 3. Results

### 3.1. Race, Ethnicity, and Genotype Distribution of the Georgia CF Population

Racial and ethnic distribution of PwCF in Georgia (*n* = 969) shows that the state is more diverse than the national U.S. CF population [30] (Figure S1). For example, 86% of individuals with CF in Georgia identify as White, compared to 91% nationally. In addition, higher percentages of PwCF in Georgia identify as Black/African American (9%) or Two or More Races (3%), but a lower percentage identify as Hispanic/Latino (~6%). Georgia’s pediatric CF population is even more diverse than the adult CF population, with ~10% identifying as Black/African American, ~5% as Two or More Races, and ~10% as Hispanic/Latino (Figure S2).

A total of 1882 *CFTR* alleles were identified from our population after excluding benign and unknown variants (as defined in Section 2.2). The most common *CFTR* genotype among PwCF in Georgia is F508del homozygous, constituting 44% of the population. However, this genotype is less frequent among all minoritized racial/ethnic groups (0–35%) compared to non-Hispanic White (50%) PwCF (Table 1). F508del heterozygotes represent 40% of the Georgia CF population, while 16% of PwCF carry one or more non-F508del variant including rare, benign, or unknown variants. Minoritized PwCF more commonly encode non-F508del variants (13–67%) compared to non-Hispanic White PwCF (12%). Furthermore, the majority of the Georgia CF population has Two or More variants identified (95%), but Black PwCF exhibit the highest rate of incomplete *CFTR* genotyping with no variants identified (6%), while Asian PwCF have the highest rate of only one variant identified (33%).

### 3.2. CFTR Variant Frequencies Among Individuals in Georgia Compared to the United States

To determine whether *CFTR* genotypic diversity differed in Georgia, the most prevalent variants identified among PwCF in the state at the individual level were compared to summary results from the entire U.S. CF population described in the 2021 CFFPR Report [30] (Table 2). National data include the 25 most common variants. The top 27 variants in Georgia are reported due to multiple variants occurring at the same frequency. Incidence of the two most common *CFTR* variants in Georgia, F508del (83.7%) and G551D (4.9%), is approximately the same across national data (85.5% or 4.2%, respectively). In contrast, G542X is the second-most prevalent variant in the U.S. (4.5%), versus seventh in Georgia (2.4%). The third-most common variant in Georgia, 3120+1G->A, ranks 12th in the country and exhibits an allele frequency nearly three times higher in the state (3.1%) compared to the U.S. (1.2%). Seven of the top twenty-seven variants in Georgia are not represented among the most prevalent variants in the nation, and nine are not included on the Luminex-39 variant panel used for GA NBS (Table 2, asterisks and double dagger, respectively).

### 3.3. Allele Frequencies in the Georgia CF Population According to Race and Ethnicity

Allele level frequencies for the 29 most common *CFTR* variants in Georgia (all variants occurring at a frequency of >5 alleles) are shown and further annotated according to race and ethnicity in Table S2. To ascertain which *CFTR* variants exhibited the highest prevalence among demographic groups, the five most common variants (other than F508del) with allele count >1 were recorded for each race and ethnicity (Table 3). F508del is uniformly the most common, but this variant exhibits a wide range of occurrence across groups (21.7–70.7%). After F508del, variants with the highest frequencies detected among minoritized populations are distinct from those observed for non-Hispanic White PwCF in Georgia. For example, 3120+1G->A is most common in non-Hispanic Black PwCF, and G542X is most common in Hispanic/Latino individuals. Among PwCF identifying as Two or More Races, over one quarter of the variants were found in only one individual in that group. It is also noteworthy that among these most common *CFTR* variants for each race/ethnicity group, eight are not included on the Luminex-39 panel currently used for CF NBS in Georgia (Table 3, double dagger). This issue disproportionately affects Hispanic/Latino patients, with four of their top five non-F508del variants omitted from the Luminex-39 panel.

### 3.4. CFTR Genetic Panel Performance in the Georgia CF Population

**Figure 1:**
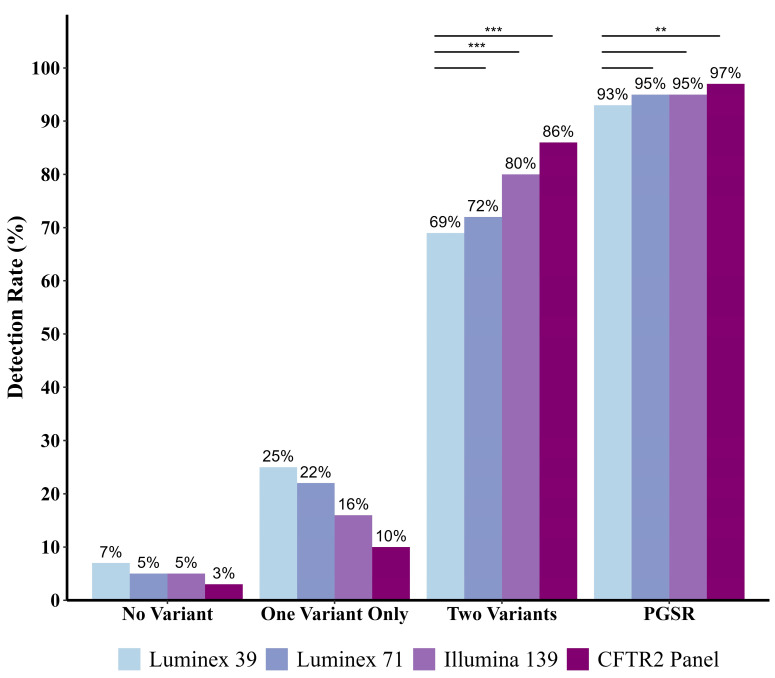
Variant detection rates for four *CFTR* variant panels tested in the Georgia CF population (*n* = 969 individuals). Pairwise comparisons of positive genetic screening rate (PGSR) were conducted between each panel and the reference, Luminex-39 (current in Georgia). Post hoc Bonferroni correction was applied to adjust for multiple comparisons. Each comparison is represented by a solid line. Statistical calculations are provided in Table S3, and significance is marked with ** (*p* < 0.01) or *** (*p* < 0.001).

To evaluate performance of genetic tests currently in use for CF NBS at identifying PwCF in Georgia, four *CFTR* panels were compared. These include the Luminex-39, Luminex-71, NGS-based Illumina-139, and a 719 variant CFTR2-based NGS panel. Detection rates for two, only one, or no *CFTR* variants are shown for each panel, along with detection of at least one variant, representing case identification (positive genetic screening rate, PGSR) (Figure 1). The CFTR2 panel performs best for PGSR, with significantly higher case detection than the Luminex-39 (97% vs. 93%; *p* = 0.002) (Figure 1, Table S3). Although PGSR improves to 95% for both the Luminex-71 and Illumina-139 panels, pairwise comparison to Luminex-39 PGSR does not show statistical significance (Figure 1, Table S3).

For Georgia NBS, identification of one *CFTR* variant prompts confirmatory sweat testing, whereas identification of two *CFTR* variants facilitates immediate referral to a CF Care Center. With increasing panel size, two-variant identification significantly improves, as evidenced by comparing detection rates of the Luminex-39 (69%) to the Illumina-139 (80%; *p* < 0.001) and CFTR2 panel (86%; *p* < 0.001) (Figure 1, Table S3). However, two-variant identification does not significantly improve using the Luminex-71, compared to the Luminex-39.

**Figure 2:**
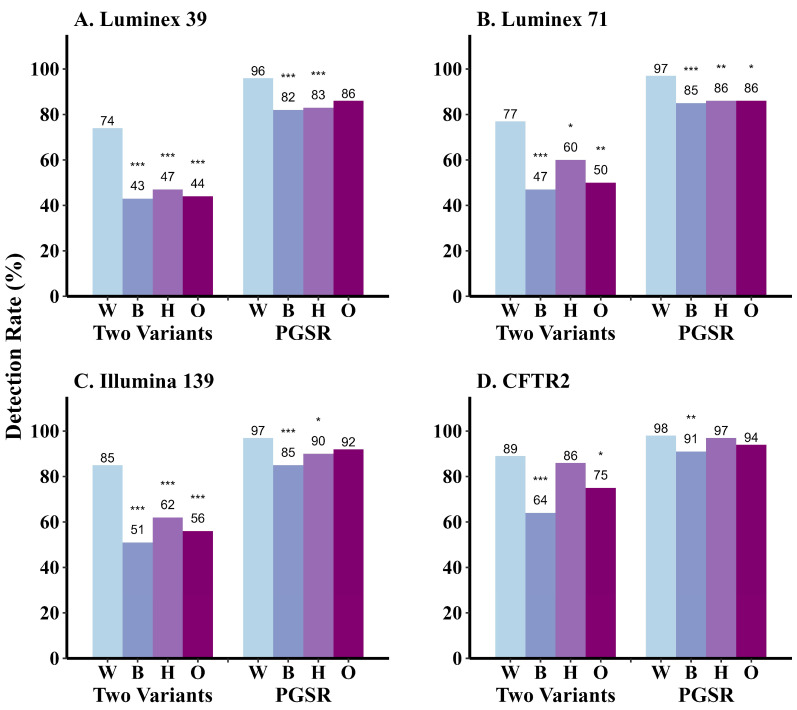
*CFTR* variant panel performance according to race and ethnicity among 969 individuals with CF receiving care in Georgia. *, *p* < 0.05; **, *p* < 0.01, *** *p* ≤ 0.001 (Pearson’s Chi-squared test with post hoc Bonferroni correction to adjust for multiple comparisons). Statistical calculations are provided in Tables S5 and S6. The overall *p*-value for detection of variants by race and ethnicity is compared to non-Hispanic White as a reference group. PGSR, Positive Genetic Screening Rate; W, White (non-Hispanic); B, Black/African American (non-Hispanic); H, Hispanic/Latino; O, Other Race (non-Hispanic). The category of Other Race (non-Hispanic) includes PwCF identifying as Two or More Races, Asian, Native Hawaiian or Other Pacific Islander, American Indian or Alaska Native, and Other race.

Each panel was also assessed for equity in variant detection rates among different racial and ethnic groups in Georgia (Figure 2, Tables S4–S6). PGSR (case detection) generally improves with increasing panel size for all groups (Figure 2). PGSR is significantly lower for Hispanic/Latino PwCF compared to non-Hispanic White individuals screened with the Luminex-39 (*p* < 0.001), Luminex-71 (*p* = 0.001), or Illumina-139 (*p* = 0.034) panels (Figure 2A–C, Table S5). For Hispanic and Other race PwCF, PGSR achieves similar levels to the non-Hispanic White cohort with the CFTR2 panel (Figure 2D). However, PGSR remains significantly lower for non-Hispanic Black PwCF compared to non-Hispanic White PwCF across all panels (*p* < 0.001; Figure 2, Table S5). In the case of CFTR2, 9% of non-Hispanic Black PwCF would not have a variant identified compared to 2% of non-Hispanic White PwCF and 3% of Hispanic PwCF, and 6% of non-Hispanic Other race PwCF (Table S4). Across all panels except the CFTR2 panel, minoritized PwCF are significantly less likely to have two variants detected compared to the non-Hispanic White cohort (Figure 2, Table S6). Using the CFTR2 panel, two-variant detection is similar for non-Hispanic White PwCF (89%) and Hispanic PwCF (86%) but remains significantly lower for Black PwCF (64%) and other Race PwCF (75%) (Figure 2D).

In late 2024, a CFTR2 database update increased the number of CF-causing variants from 719 to 1085. A hypothetical assay including these 1085 variants resulted in no additional case detection. However, two variants (instead of one) are identified in six additional individuals, five of whom are Black. This panel would increase the overall two-variant detection rate to 87%, and the two-variant detection rate among Black individuals to 70% (Table S4).

## 4. Discussion

This cross-sectional study analyzed *CFTR* variant frequency in the Georgia CF population to inform recommendations to improve equitable and early diagnosis in CF newborn screening. Our results describe the racial and ethnic diversity of the Georgia CF population, with a higher percentage of individuals identifying as Black and Two or More Races compared to the nation and a sizeable Hispanic population. The Georgia CF pediatric population is even more diverse than the adult population. This difference could be related to increasing diversity in the state, higher rates of diagnosis in the Hispanic CF population nationally over the last decade, or to a survival effect [3,27,28,30,38]. Georgia offers an important opportunity to assess performance of CF newborn screening genetic approaches in a racially and ethnically diverse state.

Examining completeness of genotyping, we observed that 5% of PwCF had zero or only one identified non-benign variant despite full *CFTR* sequencing. PwCF identifying as Black had the highest rate of incomplete *CFTR* genotyping with no identified non-benign variants (6%), while Asian PwCF had the highest rate of only one identified variant (33%). This group of individuals with incomplete genotyping presents unique considerations when contemplating the role of sweat testing in states that screen for all CF-causing variants.

Further evaluation of non-F508del *CFTR* variants showed that the variants occurring most commonly among Georgia individuals with CF were distinct compared to national U.S. data. Seven of the most frequent variants in Georgia PwCF were not represented in the national data. The presence of these variants (2789+2insA, G1061R, P67L, A559T, 2307insA, R560T, and S945L) underscores the uniqueness of the regional *CFTR* genetic landscape. We also examined the performance of Luminex-39, Georgia’s current NBS panel, for detection of the most common variants seen in our population. Of the most common CF variants in Georgia, nine are not on our current panel. Of note, 5T, D1152H, and 2789+2insA are VVCC, which are typically excluded from NBS genetic panels.

With Georgia’s larger population of individuals identifying as Black and Two or More Races, we represent a unique population with higher frequencies of some “rarer” *CFTR* variants compared to national CF data. Notably, variants that are more common in non-White races were seen at higher frequencies in the Georgia population. Several variants that are common among Georgia PwCF of minoritized race and/or ethnicity are not included on many of the small common variant panels, including the Luminex-39 panel currently used in the state’s newborn screening algorithm. For Hispanic individuals, only one of the most common non-F508del variants is tested on the Luminex-39 panel. For PwCF identifying as Other race, non-Hispanic, only two of the most common non-F508del variants are on the panel. For individuals who reported Two or More Races, only two variants could be considered “common” in their population. These findings emphasize the importance of considering variant panels that reflect the demographics of the region to ensure equitable approaches in NBS, as recommended in a newly published consensus guideline [39]. Tailoring genetic testing strategies based on regional allele variation could enhance the sensitivity and effectiveness of CF NBS.

A goal of this project was to inform recommendations for updating the genetic testing approach in Georgia’s CF NBS algorithm. To accomplish this goal, we analyzed detection rates among Georgia’s current Luminex-39 variant panel and three expanded panels that could potentially improve equity of diagnosis. As expected, case detection rates (PGSR) and two-variant detection rates rose with increasing panel size, including in PwCF of minoritized ancestries. Increasing to the largest variant panel that includes all CF-causing variants in CFTR2 would significantly improve overall PGSR from 93% to 97% and two-variant detection from 69% to 86%. However, most panels had significantly lower case detection rates for minoritized PwCF compared to non-Hispanic White PwCF. Although the CFTR2 panel appears the most equitable, PGSR and two-variant detections remained lower in PwCF identifying as non-Hispanic Black (91% and 64%, respectively). A CFTR2-based 1085 variant panel, which is recommended by the new guidelines [39], did not further increase case detection compared to the CFTR2-719 panel but improved two-variant detection to 87% and two-variant detection increased among Black individuals to 70%.

Two-variant detection on NBS leads to faster clinic referral and treatment initiation, eliminating the need for sweat chloride testing prior to establishing care. In Georgia, there are only two labs that are accredited to perform sweat chloride testing, which may hinder timely testing. Delays in sweat testing may be influenced by other social determinants of health, including primary care provider bias, access to transportation, child-care, distance from sweat testing centers, and time off work [40]. Consistent with the new guideline, our findings underscore the importance of incorporating expanded *CFTR* testing into NBS to optimize both case detection and two-variant detection and facilitate more equitable and timely diagnosis. However, higher costs and specialized expertise remain important additional considerations for states evaluating NGS-based expanded panels.

While we were conducting our study, an analysis was published by McGarry et al., which reported comparable national detection rates for Luminex-39 and Illumina-139 to our results in Georgia [11]. Of note, McGarry’s study only included fully genotyped individuals in CFFPR, whereas our study included individuals with one or more unknown variants as well as PwCF not enrolled in CFFPR for analysis. Our higher detection rates using the CFTR2 panel are due to CFTR2 updates in April 2023, which expanded the number of disease-causing variants to 719. McGarry et al. also examined performance of the Illumina-139 panel by state. Our results reveal higher detection rates for Illumina-139 in Georgia, likely due to our ability to supplement registry data with chart review for missing and imprecise genotypes. Individuals with “incomplete” genotypes were included in this study to recognize current limitations in genomic technologies and emphasize that regardless of panel size and expansive sequencing technologies, screening tests remain unable to detect all cases of CF on newborn screening [41].

Our study has several limitations. While we analyzed *CFTR* genotypes in the current Georgia CF population, the demographics of this group may differ from infants being born in Georgia at present. In fact, birth census data and the demographics in our pediatric population suggest that Georgia births are becoming increasingly diverse, emphasizing the importance of using broader *CFTR* panels in NBS. Additionally, some of the individuals receiving care in Georgia may have been born in other states or may live in surrounding states and thus may differ from current births in Georgia. Furthermore, there are likely still undiagnosed individuals living with CF, and these individuals may more likely be of minoritized races and ethnicities due to medical provider biases and biases present in current *CFTR* genetic panels. A few PwCF living in Georgia may also receive care at non-accredited CF centers and would be excluded from our dataset. Lastly, our study used race and ethnicity data collected from the CFFPR, where it is entered based on self-report at the time of patient registration.

## 5. Conclusions

This study describes *CFTR* variant frequency in the diverse Georgia CF population and demonstrates that variant detection using common NBS panels will be lower among PwCF in Georgia of minoritized races and ethnicities. Use of expanded, NGS-based *CFTR* panels would improve equity in CF diagnosis by newborn screening in Georgia, thereby reducing health disparities resulting from missed and delayed diagnosis.

## Figures and Tables

**Table 1:** *CFTR* genotype characteristics of the Georgia CF population according to race and ethnicity.

	Overall	White, Non-Hispanic	Black or African American, Non-Hispanic	Hispanic or Latino, Any Race	Two or More Races, Non-Hispanic	Asian, Non-Hispanic	Other, Non-Hispanic
	*n* = 969	*n* = 788	*n* = 87	*n* = 58	*n* = 23	*n* = 9	*n* = 4
**F508del variant prevalence**							
F508del homozygotes	424 (44%)	394 (50%)	8 (9%)	14 (24%)	8 (35%)	0 (0%)	0 (0%)
F508del heterozygotes	387 (40%)	297 (38%)	46 (53%)	27 (47%)	12 (52%)	3 (33%)	2 (50%)
No F508del variant	158 (16%)	97 (12%)	33 (38%)	17 (29%)	3 (13%)	6 (67%)	2 (50%)
**Genotyping completeness**							
Two or more known variants	921 (95%)	754 (96%)	77 (89%)	57 (98%)	23 (100%)	6 (67%)	4 (100%)
Only one known variant	31 (3%)	22 (3%)	5 (6%)	1 (2%)	0 (0%)	3 (33%)	0 (0%)
Unknown variants	17 (2%)	12 (2%)	5 (6%)	0 (0%)	0 (0%)	0 (0%)	0 (0%)

Values indicate *n* (column %). Variants classified as benign polymorphisms are categorized as unknown.

**Table 2:** Individual level *CFTR* variant prevalence among PwCF in Georgia (*n* = 969) compared to the U.S. CF population.

Most Common *CFTR* Variants Among PwCF in the U.S. [30]		Most Common *CFTR* Variants Among PwCF in Georgia	
Legacy Name	*n* (%) of Individuals	Legacy Name	*n* (%) of Individuals
F508del	27,269 (85.5%)	F508del	811 (83.7%)
G542X	1443 (4.5%)	G551D	47 (4.9%)
G551D	1352 (4.2%)	3120+1G->A	30 (3.1%)
R117H	1048 (3.3%)	R117H	29 (3.0%)
N1303K	752 (2.4%)	621+1G->T	25 (2.6%)
W1282X	708 (2.2%)	G542X	23 (2.4%)
3849+10kbC->T	588 (1.8%)	N1303K	21 (2.2%)
R553X	557 (1.7%)	2789+2insA *^^‡^	19 (2.0%)
621+1G->T	499 (1.6%)	5T ^^‡^	17 (1.8%)
1717-1G->A	497 (1.6%)	R553X	17 (1.8%)
2789+5G->A	474 (1.5%)	W1282X	16 (1.7%)
3120+1G->A	396 (1.2%)	3849+10kbC->T	13 (1.3%)
D1152H	343 (1.1%)	2184insA ^‡^	11 (1.1%)
5T	338 (1.1%)	1717-1G->A	10 (1.0%)
3272-26A->G	257 (0.8%)	L206W ^‡^	10 (1.0%)
2184insA	252 (0.8%)	I507del	9 (0.9%)
R1162X	248 (0.8%)	3272-26A->G ^‡^	8 (0.8%)
I507del	237 (0.7%)	G1061R *^‡^	8 (0.8%)
G85E	225 (0.7%)	P67L *^‡^	8 (0.8%)
L206W	220 (0.7%)	A559T *	7 (0.7%)
3659delC	219 (0.7%)	D1152H ^^‡^	7 (0.7%)
1898+1G->A	214 (0.7%)	1898+1G->A	6 (0.6%)
R334W	200 (0.6%)	2789+5G->A	6 (0.6%)
R347P	197 (0.6%)	2307insA *	6 (0.6%)
A455E	189 (0.6%)	R1162X	6 (0.6%)
--		R560T *	6 (0.6%)
--		S945L *^‡^	6 (0.6%)

Variants classified as benign polymorphisms are excluded. The number and percentage of individuals with a given variant include those with one or two copies of the variant. Individuals may be included multiple times as each variant is counted. *, not present among the 25 most common variants reported for the U.S. CF population. ^, variant of varying clinical consequence (VVCC) according to CFTR2. ^‡^, not included on the Luminex-39 panel.

**Table 3:** Most common *CFTR* variants according to race/ethnicity on the allele level in Georgia, occurring at a frequency of >1.

White, Non-Hispanic		Black or African American, Non-Hispanic		Hispanic or Latino		Two or More Races, Non-Hispanic		Other Race, Non-Hispanic ^a^	
*n* (Variants) = 1535		*n* (Variants) = 163		*n* (Variants) = 115		*n* (Variants) = 46		*n* (Variants) = 23	
Variant	*n* (%)	Variant	*n* (%)	Variant	*n* (%)	Variant	*n* (%)	Variant	*n* (%)
F508del	1085 (70.7%)	F508del	62 (38.0%)	F508del	55 (47.8%)	F508del	28 (60.9%)	F508del	5 (21.7%)
G551D	45 (2.9%)	3120+1G->A	20 (12.3%)	G542X	6 (5.2%)	3120+1G->A	5 (10.9%)	V456A ^‡^*	3 (13.0%)
R117H	28 (1.8%)	A559T	5 (3.1%)	3272-26A->G ^‡^	4 (3.5%)			S549N *	2 (8.7%)
621+1G->T	24 (1.6%)	2307insA	5 (3.1%)	L206W ^‡^	4 (3.5%)			R334W	2 (8.7%)
N1303K	19 (1.2%)	I618T ^‡^*	4 (2.5%)	1811+1G->A ^‡^*	4 (3.5%)			R709X ^‡^*	2 (8.7%)
2789+2insA ^‡^^	19 (1.2%)	S549N *	3 (1.8%)	A559P ^‡^*^+^	4 (3.5%)				

Variants classified in the CFTR2 database as benign polymorphisms are excluded. Many variants in the Two or More races, non-Hispanic group occurred in only one individual in the group, thus only two variants are listed. *, not present among the most common variants in Georgia (Table S2). ^‡^, not included on the Luminex-39 panel. ^, Variant of varying clinical consequence (VVCC) according to CFTR2 [32,34]. ^+^, not listed in CFTR2, classified as Likely Pathogenic (2 laboratories) and variant of unknown significance (1 laboratory) in ClinVar database, per guidelines of the American College of Medical Genetics and Genomics [36,37]. ^a^, includes individuals identifying as non-Hispanic Asian or Other race.

## Data Availability

Aggregated data available by reasonable request to the corresponding author.

## Supplementary Materials

The following supporting information can be downloaded at: https://www.mdpi.com/article/10.3390/ijns11040085/s1, Table S1: Legacy names and corresponding Human Genome Variation Society nomenclature for *CFTR* variants described in this study; Table S2: Allele level prevalence for the 29 most common *CFTR* variants (occurring at a frequency of >5 alleles) found in the PwCF in Georgia and distribution according to race/ethnicity; Table S3: Statistical analysis of *CFTR* genetic panel performance in the Georgia CF population; Table S4: Number of *CFTR* variants detected on each panel by race/ethnicity; Table S5: Pairwise comparisons for positive genetic screening rate by race/ethnicity for each *CFTR* panel; Table S6: Pairwise comparisons for two variant detection vs. one or no variant detection rate by race/ethnicity for each *CFTR* panel.; Figure S1: Race and ethnicity of people with CF in Georgia compared to the U.S. CF population; Figure S2: Race and ethnicity of children with CF in Georgia.

## Abbreviations

CF	cystic fibrosis
*CFTR*	cystic fibrosis transmembrane conductance regulator
CFTR2	Clinical and Functional Translation of CFTR
NBS	newborn screening
IRT	immunoreactive trypsinogen
PwCF	people with cystic fibrosis
CFF	Cystic Fibrosis Foundation
CFFPR	Cystic Fibrosis Foundation Patient Registry
NGS	next-generation sequencing
PGSR	positive genetic screening rate
U.S.	United States
VVCC	variant of varying clinical consequence
ACMG	American College of Medical Genetics and Genomics
