# Supplementary material for: CFTR Variant Frequencies and Newborn Screening Panel Performance in the Diverse CF Population Receiving Care in the State of Georgia
Source: Int J Neonatal Screen. 2025 Sep 26;11(4):85. doi: 10.3390/ijns11040085 (PMC12551088; doi:10.3390/ijns11040085)
Supplement: Supplementary file 1 [file IJNS-11-00085-s001.zip › IJNS-3739900-supplementary.pdf]

SUPPLEMENTARY MATERIAL FOR:

CFTR Variant Frequencies and Newborn Screening Panel Performance in the Diverse CF Population Receiving Care in the State of Georgia

Eileen Barr [1,2], Brittany Truitt [2,3], Andrew Jergel [3], Shasha Bai [3], Kathleen McKie [4], Rossana Sanchez Russo [1], Kathryn E. Oliver [3], and Rachel W. Linnemann [2,3*]

[1]Department of Human Genetics, Emory University, Atlanta, GA, 30322, USA

[2]Children's Healthcare of Atlanta, Atlanta, GA, 30329, USA

[3]Department of Pediatrics, Emory University, Atlanta, GA, 30322, USA

[4]Department of Pediatrics, Augusta University, Augusta, GA, 30912, USA

Correspondence: rachel.linnemann@emory.edu, Tel.: +1-404-785-5437

LIST OF SUPPLEMENTARY TABLES AND FIGURES

Table S1: Legacy names and corresponding Human Genome Variation Society nomenclature for *CFTR* variants described in this study.
Table S2: Allele level prevalence for the 29 most common *CFTR* variants (occurring at a frequency of >5 alleles) found in the PwCF in Georgia and distribution according to race/ethnicity
Table S3: Statistical analysis of *CFTR* genetic panel performance in the Georgia CF population
Table S4: Number of *CFTR* variants detected on each panel by race/ethnicity
Table S5: Pairwise comparisons for positive genetic screening rate by race/ethnicity for each *CFTR* panel
Table S6: Pairwise comparisons for two variant detection vs. one or no variant detection rate by race/ethnicity for each *CFTR* panel.
Figure S1: Race and ethnicity of people with CF in Georgia compared to the U.S. CF population.
Figure S2: Race and ethnicity of children with CF in Georgia.

Table S1. Legacy names and corresponding Human Genome Variation Society nomenclature for *CFTR* variants described in this study.

**Legacy Name**	**Amino acid (p.)**	**Nucleotide (c.)**
1717-1G->A	p.?	c.1585-1G>A
1811+1G->A	p.?	c.1679+1G>A
1898+1G->A	p.?	c.1766+1G>A
2184insA	p.Gln685ThrfsX4	c.2052_2053insA
2307insA	p.Glu726ArgfsX4	c.2175_2176insA
2789+2insA	p.?	c.2657+2_2657+3insA
2789+5G->A	p.?	c.2657+5G>A
3120+1G->A	p.?	c.2988+1G>A
3272-26A->G	p.?	c.3140-26A>G
3659delC	p.Lys1177SerfsX15	c.3528delC
3849+10kbC->T	p.?	c.3718-2477C>T
5T/7T/9T	p.?	c.1210-12_1210-6T [(5-9)]
621+1G->T	p.?	c.489+1G>T
A455E	p.Ala455Glu	c.1364C>A
A559P	p.Ala559Pro	c.1675G>C
A559T	p.Ala559Thr	c.1675G>A
D1152H	p.Asp1152His	c.3454G>C
F508del	p.Phe508del	c.1521_1523del
G1061R	p.Gly1061Arg	c.3181G>C
G542X	p.Gly542X	c.1624G>T
G551D	p.Gly551Asp	c.1652G>A
G85E	p.Gly85Glu	c.254G>A
I507del	p.Ile507del	c.1519_1521del
I618T	p.Ile618Thr	c.1853T>C
L206W	p.Leu206Trp	c.617T>G
M470V	p.Met470Val	c.1408A>G
N1303K	p.Asn1303Lys	c.3909C>G
P67L	p.Pro67Leu	c.200C>T
R1162X	p.Arg1162X	c.3484C>T
R117H	p.Arg117His	c.350G>A
R334W	p.Arg334Trp	c.1000C>T
R347P	p.Arg347Pro	c.1040G>C
R553X	p.Arg553X	c.1657C>T
R560T	p.Arg560Thr	c.1679G>C
R709X	p.Arg709X	c.2125C>T
S341P	p.Ser341Pro	c.1021T>C
S549N	p.Ser549Asn	c.1646G>A
S945L	p.Ser945Leu	c.2834C>T
T854T	p.Thr854Thr	c.2562T>C or c.2562T>G or c.2562T>A
V456A	p.Val456Ala	c.1367T>C
W1282X	p.Trp1282X	c.3846G>A

Table S2. Allele level prevalence for the 29 most common *CFTR* variants (occurring at a frequency of >5 alleles) found in the PwCF in Georgia and distribution according to race/ethnicity

***CFTR* variant**	**Overall**	**White, non-Hispanic**	**Black or African American, non-Hispanic**	**Hispanic or Latino**	**Two or More Races, non-Hispanic**	**Other Race, non-Hispanic**
	N = 1,882	N = 1,535	N = 163	N = 115	N =46	N = 23
F508del	1,235 (65.6%)	1,085 (70.7%)	62 (38.0%)	55 (47.8%)	28 (60.9%)	5 (20.8%)
G551D	48 (2.6%)	45 (2.9%)	2 (1.2%)	0 (0.0%)	1 (2.2%)	0 (0.0%)
3120+1G->A	30 (1.6%)	2 (0.1%)	20 (12.3%)	3 (2.6%)	5 (10.9%)	0 (0.0%)
R117H	29 (1.5%)	28 (1.8%)	0 (0.0%)	0 (0.0%)	0 (0.0%)	1 (4.3%)
621+1G->T	25 (1.3%)	24 (1.6%)	1 (0.6%)	0 (0.0%)	0 (0.0%)	0 (0.0%)
G542X	24 (1.3%)	17 (1.1%)	1 (0.6%)	6 (5.2%)	0 (0.0%)	0 (0.0%)
N1303K	21 (1.1%)	19 (1.2%)	1 (0.6%)	1 (0.9%)	0 (0.0%)	0 (0.0%)
2789+2insA	19 (1.0%)	19 (1.2%)	0 (0.0%)	0 (0.0%)	0 (0.0%)	0 (0.0%)
5T	18 (1.0%)	17 (1.1%)	1 (0.6%)	0 (0.0%)	0 (0.0%)	0 (0.0%)
R553X	17 (0.9%)	16 (1.0%)	0 (0.0%)	1 (0.9%)	0 (0.0%)	0 (0.0%)
W1282X	17 (0.9%)	17 (1.1%)	0 (0.0%)	0 (0.0%)	0 (0.0%)	0 (0.0%)
3849+10kbC->T	13 (0.7%)	12 (0.8%)	0 (0.0%)	1 (0.9%)	0 (0.0%)	0 (0.0%)
2184insA	11 (0.6%)	11 (0.7%)	0 (0.0%)	0 (0.0%)	0 (0.0%)	0 (0.0%)
1717-1G->A	10 (0.5%)	9 (0.6%)	1 (0.6%)	0 (0.0%)	0 (0.0%)	0 (0.0%)
3272-26A->G	10 (0.5%)	6 (0.4%)	0 (0.0%)	4 (3.5%)	0 (0.0%)	0 (0.0%)
L206W	10 (0.5%)	6 (0.4%)	0 (0.0%)	4 (3.5%)	0 (0.0%)	0 (0.0%)
I507del	9 (0.5%)	7 (0.5%)	0 (0.0%)	2 (1.7%)	0 (0.0%)	0 (0.0%)
G1061R	8 (0.4%)	6 (0.4%)	2 (1.2%)	0 (0.0%)	0 (0.0%)	0 (0.0%)
P67L	8 (0.4%)	7 (0.5%)	0 (0.0%)	0 (0.0%)	0 (0.0%)	1 (4.3%)
A559T	7 (0.4%)	0 (0.0%)	5 (3.1%)	2 (1.7%)	0 (0.0%)	0 (0.0%)
D1152H	7 (0.4%)	4 (0.3%)	0 (0.0%)	3 (2.6%)	0 (0.0%)	0 (0.0%)
1898+1G->A	6 (0.3%)	6 (0.4%)	0 (0.0%)	0 (0.0%)	0 (0.0%)	0 (0.0%)
2307insA	6 (0.3%)	0 (0.0%)	5 (3.1%)	0 (0.0%)	1 (2.2%)	0 (0.0%)
2789+5G->A	6 (0.3%)	6 (0.4%)	0 (0.0%)	0 (0.0%)	0 (0.0%)	0 (0.0%)
R1162X	6 (0.3%)	5 (0.3%)	1 (0.6%)	0 (0.0%)	0 (0.0%)	0 (0.0%)
R334W	6 (0.3%)	2 (0.1%)	2 (1.2%)	0 (0.0%)	0 (0.0%)	2 (8.7%)
R560T	6 (0.3%)	5 (0.3%)	1 (0.6%)	0 (0.0%)	0 (0.0%)	0 (0.0%)
S341P	6 (0.3%)	6 (0.4%)	0 (0.0%)	0 (0.0%)	0 (0.0%)	0 (0.0%)
S945L	6 (0.3%)	6 (0.4%)	0 (0.0%)	0 (0.0%)	0 (0.0%)	0 (0.0%)

Values shown are "n" (column %). Variants classified as benign polymorphisms are excluded. If an individual is homozygous for a variant, that variant will be counted twice in this allele level analysis.

Table S3. Statistical analysis of *CFTR* genetic panel performance in the Georgia CF population

	**Luminex-39** vs. **Luminex-71**	**Luminex-39** vs. **Illumina-139**	**Luminex-39** vs. **CFTR2-719**
One variant vs. two variant detection	>0.999	**<0.001**	**<0.001**
One or no variant vs. two variant detection[1]	0.302	**<0.001**	**<0.001**
PGSR[1]	>0.999	0.344	**0.002**

Pearson's Chi-squared tests were performed and adjusted for multiple comparisons with Bonferroni correction. Comparisons that reached statistical significance ($p < 0.05$) are bolded. PGSR, positive genetic screening rate. [1]Represented graphically in Figure 1.

Table S4. Number of *CFTR* variants detected on each panel by race/ethnicity.

	**Overall**	**White, non-Hispanic**	**Black or African American, non-Hispanic**	**Hispanic or Latino**	**Other Race, non-Hispanic**
	N = 969	N = 788	N = 87	N = 58	N = 36
**Luminex-39**					
No Variants	63 (7%)	32 (4%)	16 (18%)	10 (17%)	5 (14%)
One Variant	241 (25%)	172 (22%)	34 (39%)	21 (36%)	15 (42%)
Two Variants	665 (69%)	584 (74%)	37 (43%)	27 (47%)	16 (44%)
**Luminex-71**					
No Variants	53 (5%)	27 (3%)	13 (15%)	8 (14%)	5 (14%)
One Variant	218 (22%)	157 (20%)	33 (38%)	15 (26%)	13 (36%)
Two Variants	698 (72%)	604 (77%)	41 (47%)	35 (60%)	18 (50%)
**Illumina-139**					
No Variants	46 (5%)	24 (3%)	13 (15%)	6 (10%)	3 (8%)
One Variant	151 (16%)	92 (12%)	30 (34%)	16 (28%)	13 (36%)
Two Variants	772 (80%)	672 (85%)	44 (51%)	36 (62%)	20 (56%)
**CFTR2-719 Panel**					
No Variants	30 (3%)	18 (2%)	8 (9%)	2 (3%)	2 (6%)
One Variant	101 (10%)	65 (8%)	23 (26%)	6 (10%)	7 (19%)
Two Variants	838 (86%)	705 (89%)	56 (64%)	50 (86%)	27 (75%)
**CFTR2-1085 Panel**					
No Variants	30 (3%)	18 (2%)	8 (9%)	2 (3%)	2 (6%)
One Variant	95 (10%)	64 (8%)	18 (21%)	6 (10%)	7 (19%)
Two Variants	844 (87%)	706 (90%)	61 (70%)	50 (86%)	27 (75%)

Values are "n" (column %). Individuals with two or more variants identified are included in the two-variant detection group.

Table S5. Pairwise comparisons for positive genetic screening rate by race/ethnicity for each *CFTR* panel.

**Panel**	**White** vs. **Black or African American**	**White** vs. **Hispanic or Latino**	**White** vs. **Other Race**
Luminex-39	**<0.001**	**<0.001**	0.053
Luminex-71	**<0.001**	**0.001**	**0.019**
Illumina-139	**<0.001**	**0.034**	0.619
CFTR2-719	**0.003**	>0.999	>0.999

Pearson's Chi-squared tests were performed and adjusted for multiple comparisons with Bonferroni correction. Comparisons that reached statistical significance ($p < 0.05$) are bolded.

Table S6. Pairwise comparisons for two variant detection vs. one or no variant detection rate by race/ethnicity for each *CFTR* panel.

**Panel**	**White** vs. **Black or African American**	**White** vs. **Hispanic or Latino**	**White** vs. **Other Race**
Luminex-39	**<0.001**	**<0.001**	**<0.001**
Luminex-71	**<0.001**	**0.024**	**0.002**
Illumina-139	**<0.001**	**<0.001**	**<0.001**
CFTR2-719	**<0.001**	>0.999	**0.046**

Pearson's Chi-squared tests were performed and adjusted for multiple comparisons with Bonferroni correction. Comparisons that reached statistical significance ($p < 0.05$) are bolded.

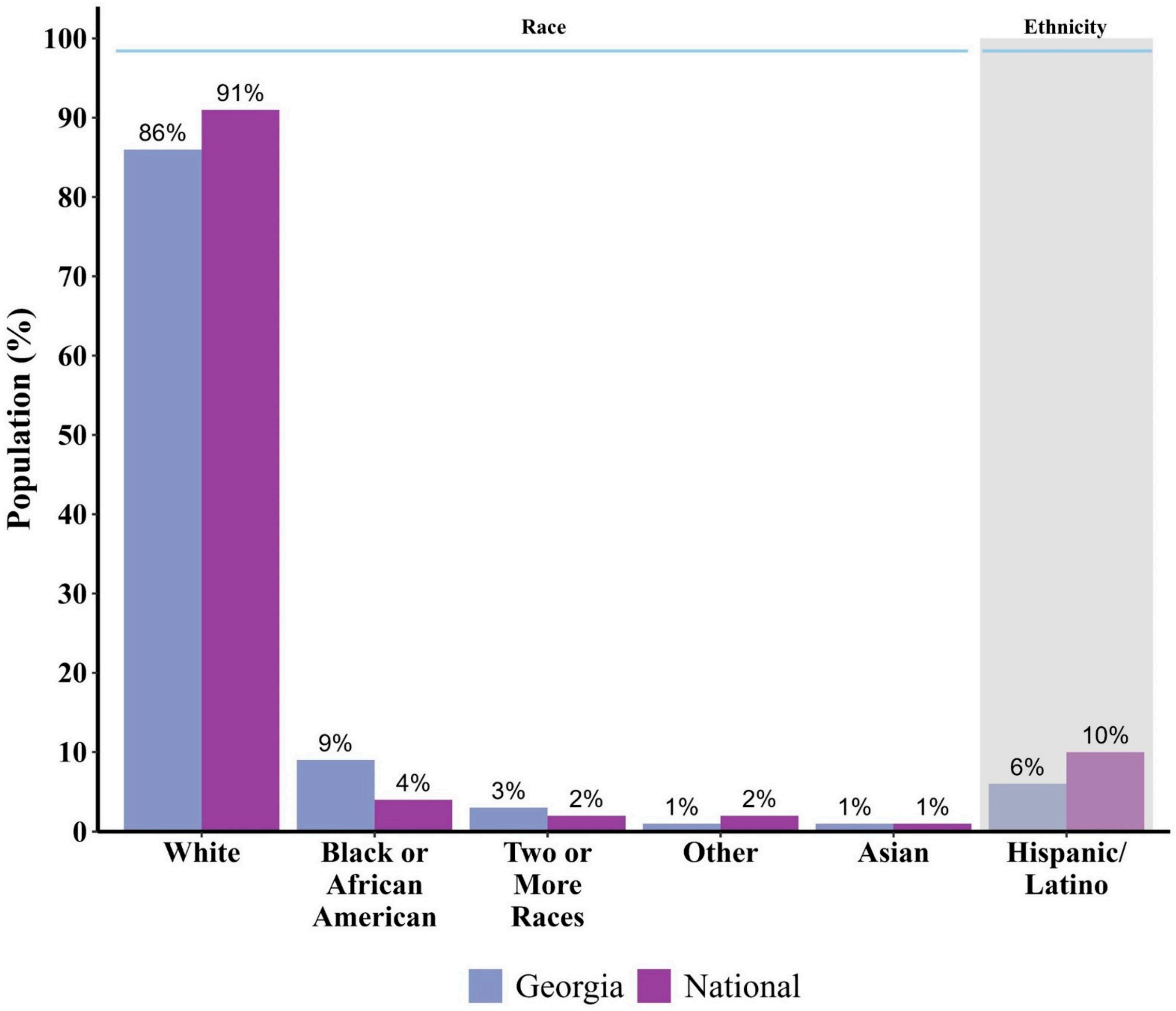

Figure S1. Race and ethnicity of people with CF in Georgia compared to the U.S. CF population. National data were obtained from the 2021 U.S. CF Foundation Patient Registry Report. "Other" includes Native Hawaiian or Other Pacific Islander and American Indian or Alaska Native.

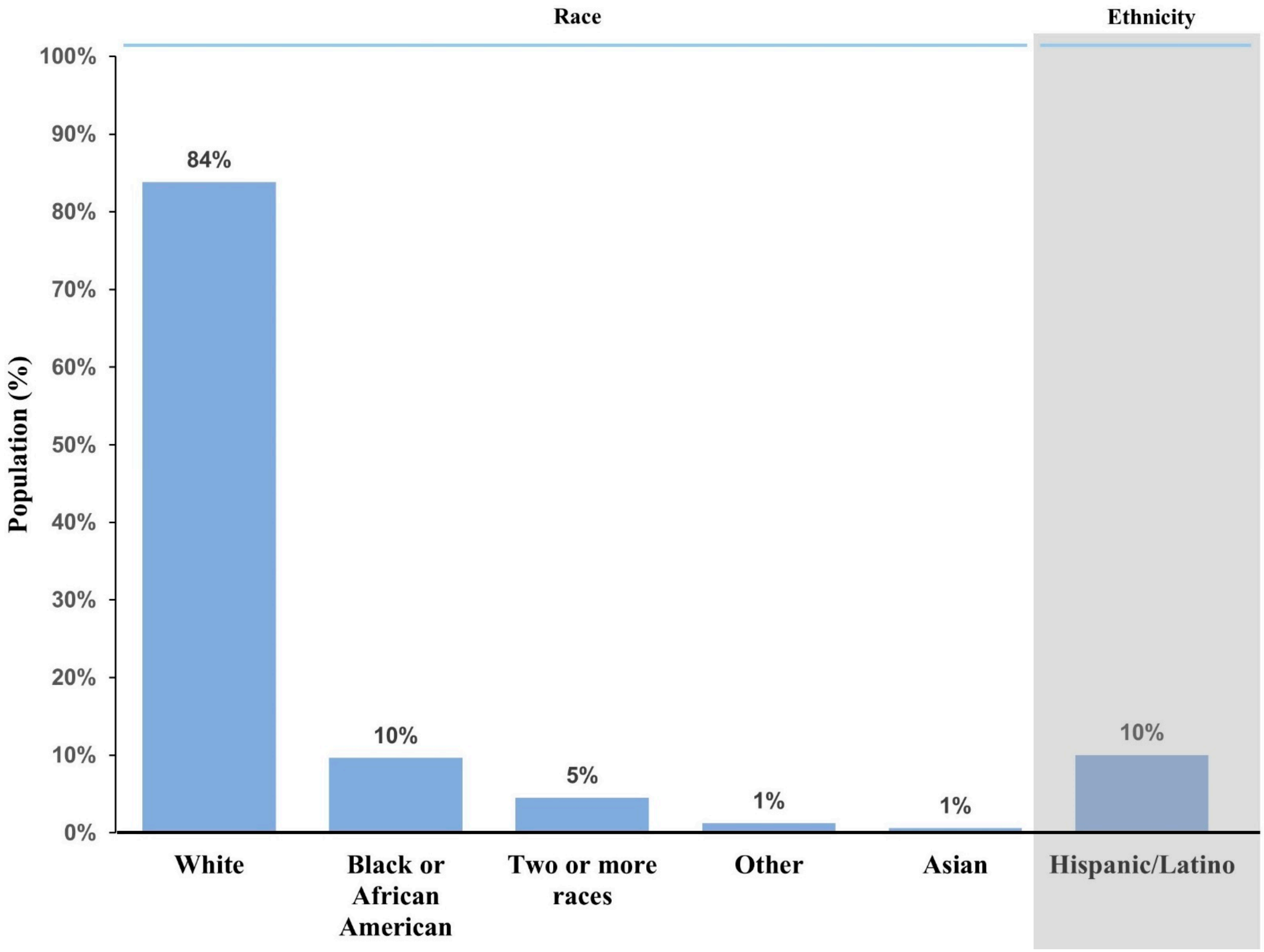

Figure S2. Race and ethnicity of children with CF in Georgia. "Other" includes Native Hawaiian or Other Pacific Islander and American Indian or Alaska Native.
